# Efficacy of lymph node dissection around the inferior mesenteric artery with preservation of the left colic artery for rectal cancer

**DOI:** 10.1002/ags3.12869

**Published:** 2024-10-11

**Authors:** Hidekazu Takahashi, Kazuhiro Saso, Masayuki Ohue, Shingo Noura, Tsukasa Tanida, Takamichi Komori, Mitsuyoshi Tei, Yoshinori Kagawa, Shunji Morita, Shu Okamura, Masakazu Miyake, Norikatsu Miyoshi, Mamoru Uemura, Makoto Fujii, Yuko Ohno, Hirofumi Yamamoto, Kohei Murata, Yuichiro Doki, Hidetoshi Eguchi, Clinical Study Group of Osaka University, Colorectal Cancer Treatment Group (CSGOCG)

**Affiliations:** ^1^ Department of Gastroenterological Surgery Osaka University Graduate School of Medicine Suita Osaka Japan; ^2^ Department of Gastroenterological Surgery Osaka International Cancer Institute Osaka Japan; ^3^ Department of Surgery Toyonaka Municipal Hospital Toyonaka Osaka Japan; ^4^ Department of Gastroenterological Surgery Higashiosaka City Medical Center Higashiosaka Osaka Japan; ^5^ Department of Gastroenterological Surgery Osaka Rosai Hospital Sakai Osaka Japan; ^6^ Department of Gastroenterological Surgery Osaka General Medical Center Osaka Japan; ^7^ Department of Surgery Itami City Hospital Itami Hyogo Japan; ^8^ Department of Gastroenterological Surgery Suita Municipal Hospital Suita Osaka Japan; ^9^ Department of Gastroenterological Surgery Rinku General Medical Center Izumisano Osaka Japan; ^10^ Division of Health Science Osaka University Graduate School of Medicine Suita Osaka Japan; ^11^ Division of Health Sciences, Department of Molecular Pathology, Graduate School of Medicine Osaka University Suita Osaka Japan; ^12^ Department of Gastroenterological Surgery Kansai Rosai Hospital Amagasaki Hyogo Japan

**Keywords:** anastomotic leak, Japanese D3, left colic artery, lymph node dissection, rectal cancer

## Abstract

**Objective:**

We investigated how Japanese D3 dissection with left colic artery (LCA) preservation affects anastomotic leakage after anterior resection with anastomosis for rectal cancer, based on the leak rate. The correlation between LCA preservation, survival, and cancer recurrence after resection was also analyzed.

**Summary and Background Data:**

It remains unclear how LCA preservation affects the anastomotic leak rate and oncological outcomes after resection remains unclear. Some reports suggested that anastomotic leakage increases local recurrence and decreases cancer‐specific survival.

**Methods:**

In this study, we enrolled and analyzed 457 patients who underwent radical resection of rectal cancer in the period October 2011 through December 2016. The attending surgeon decided preoperatively and registered whether to preserve the LCA. This trial was registered under the UMIN‐CTR Identifier UMIN000006160.

**Results:**

D3 with LCA preservation was successfully completed in 218 (89.3%) of the 244 patients registered in this group, whereas D3 without LCA preservation was successfully completed in all 213 patients registered in this group. After propensity score matching, the anastomotic leakage rate was 7.86% (11/140) after D3 with LCA preservation and 7.14% (10/140) after D3 without LCA preservation. The overall survival rates were 90.1% and 89.3%, and the recurrence‐free survival rates were 77.6% and 77.3%, respectively.

**Conclusions:**

Our findings suggest that LCA preservation has no effect on the incidence of anastomotic leakage after rectal resection with anastomosis using DST and that oncological outcomes may not be affected.

## Graphical abstract

This study examined the effect of preserving the left colic artery (LCA) during Japanese D3 dissection on anastomotic leakage and oncological outcomes in rectal cancer patients. Findings showed no significant difference in leak rate or survival between D3 with LCA preservation and D3 without LCA preservation, highlighting the need for clearer guidelines on LCA preservation criteria.

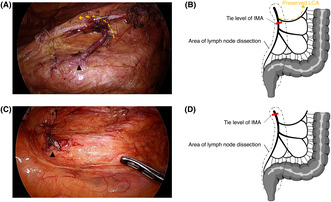

## 1 INTRODUCTION

Colorectal cancer (CRC) is the third most common malignancy and the fourth leading cause of cancer‐related mortality worldwide.^1^, ^2^, ^3^ The majority of rectal cancer cases are surgically treated with curative intent. Total mesorectal excision (TME) is the gold standard surgical resection of rectal cancer standard.^4^

Proximal D3 lymph node dissection requires accurate dissection of lymph nodes around the inferior mesenteric artery (IMA). This is similar to the complete mesocolic excision (CME) plus central vascular ligation (CVL) reported from Erlangen, Germany.^5^, ^6^ The clinical significance of CME + CVL for colon cancer has been demonstrated in a large population‐based study.^7^ Furthermore, CME + CVL and Japanese D3 dissection have been proven to be equivalent in terms of the dissection plane and central dissection, except in the intestinal resection length.^8^ From another perspective, the American Joint Committee on Cancer and International Union Against Cancer recommends the retrieval of at least 12 lymph nodes for accurate staging.^8^, ^9^ Summarizing these reports, it is speculated that accurate Japanese D3 dissection, or CME with excision at the IMA root, is ideal for more accurate staging and increasing the cancer cure rate.

Supporting this hypothesis, several studies have demonstrated the importance of lymph node dissection up to the IMA root, in terms of better survival and precise staging.^8^, ^10^, ^11^, ^12^, ^13^, ^14^, ^15^ Many of these reports describe dissection of the IMA at the root as “high tie.” However, a high tie of the IMA can reportedly decrease blood flow to the proximal colon stump^16^, ^17^, ^18^ and may have adverse effects on anastomosis, leading some to advocate a low tie with left colic artery (LCA) preservation. Indeed, LCA‐preserving low ties maintain good blood flow at the anastomotic site and are considered beneficial for anastomosis, but surgeons have reported that LCA preservation may lead to greater anastomotic tension at the anastomotic site.^19^, ^20^

Because it remains uncertain whether impaired blood flow increases the leak rate, some surgeons prefer to perform lymph node dissection up to the IMA root with IMA and LCA preservation. However, this approach is time consuming and technically demanding. We previously reported an easy and secure method for dissecting the lymph nodes around the IMA with IMA and LCA preservation (Japanese D3 with LCA preservation) via laparoscopic surgery,^21^ and have also applied this technique in open surgery. This procedure maintains blood flow at the anastomotic site while improving the accuracy of tumor staging through radical lymph node dissection.

In this multicenter, interventional, nonrandomized, open‐label study, we aimed to investigate how Japanese D3 with LCA preservation affects anastomotic leakage after anterior resection with anastomosis for rectal cancer. Leak rate was the primary outcome. To analyze the effect of LCA preservation on anastomotic leakage more sensitively, the mode of reconstruction was limited to the double‐stapler technique (DST), which is the standard anastomosis method. The secondary endpoints were overall and cancer‐specific survival, and cancer recurrence after resection. Propensity score matching (PSM) analysis was performed to obtain more accurate conclusions.

## 2 METHODS

### 2.1 Study design

**FIGURE 1:**
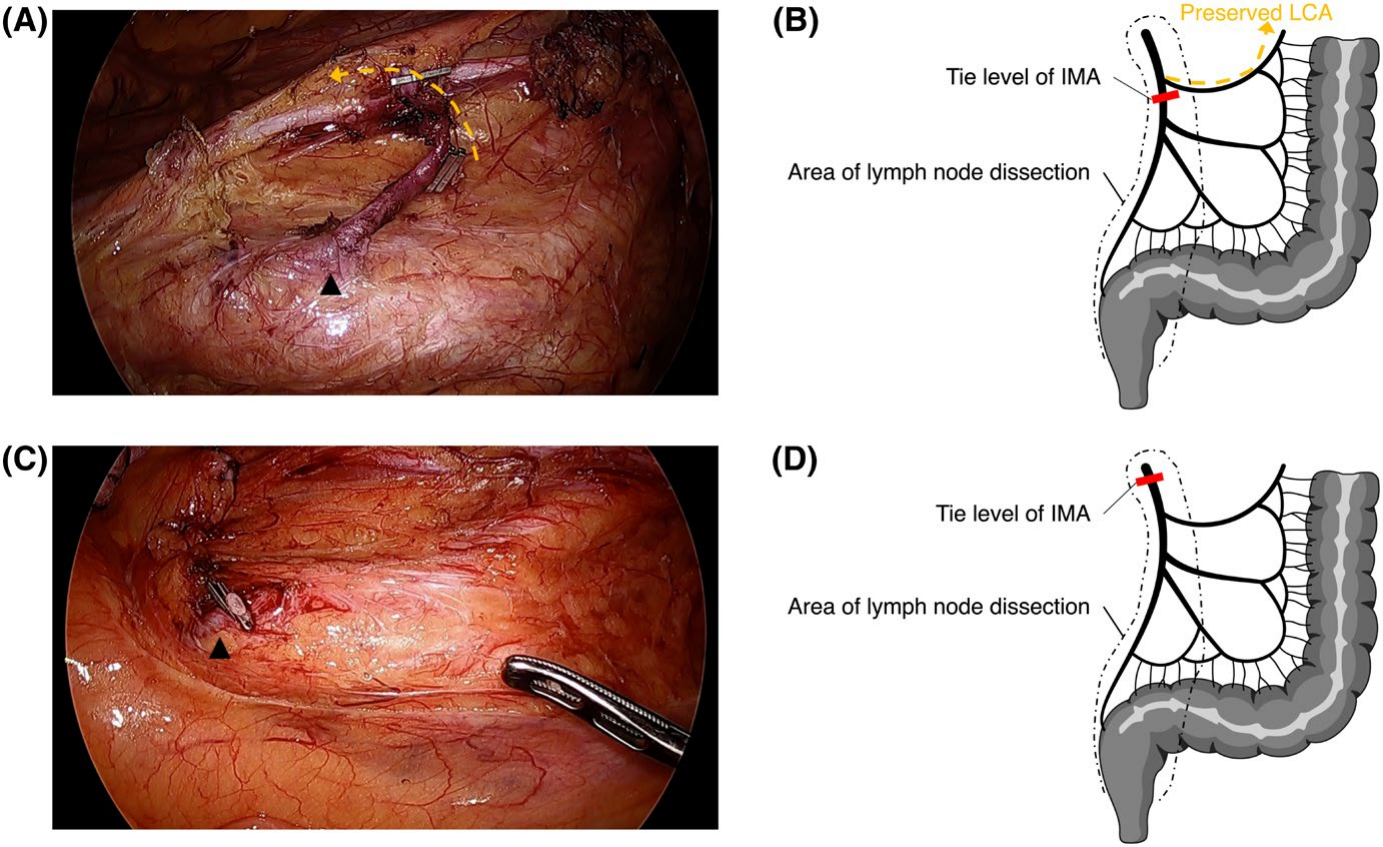
Surgical field and schema of D3 with LCA preservation and D3 without LCA preservation. (A) Representative surgical field after Japanese D3 with LCA preservation. Arrowhead indicates the root of the inferior mesenteric artery (IMA). Dashed arrow indicates the preserved left colic artery (LCA). (B) Schema of Japanese D3 with LCA preservation. (C) Representative surgical field after Japanese D3 without LCA preservation. Arrowhead indicates the root of the IMA. (D) Schema of Japanese D3 without LCA preservation.

This was a non‐randomized prospective cohort study involving 26 cancer patients. LCA preservation (Figure 1A,B) or non‐preservation (Figure 1C,D) was determined based on the attending surgeon's judgment at preoperative registration. As propensity score matching was planned after the end of enrollment, this was a post hoc analysis of a prospective study. This study was registered with UMIN‐CTR (UMIN000006160). The study protocol was consistent with the principles of the Declaration of Helsinki and approved by the ethics committees of each hospital.

### 2.2 Eligibility criteria

To be eligible for study participation, patients were diagnosed with histologically proven rectal adenocarcinoma located within 15 cm of the anal verge, classified as T2 or higher, of any N grade, or M0, according to the TNM Classification of Malignant Tumors, 7th edition. Eligible patients were over 20 years old at the time of enrollment and provided written informed consent. For inclusion, patients had to undergo elective low anterior resection with lymph node dissection around the IMA root and no prior operation involving cutting of the IMA. Finally, within 2 weeks before enrollment, eligible patients had to meet the following clinical test values: white blood cell counts ≥3000/mm^3^, platelets ≥100 000/mm^3^, total bilirubin <2.0 mg/dL, AST・ALT <100 IU/L, and serum creatinine <1.5 mg/dL.

### 2.3 Exclusion criteria

Patients were excluded if they had multiple active primaries with a disease‐free interval of less than 5 years. Patients were permitted to enroll if they had basal cell skin cancer, cervical cancer, gastric cancer, esophageal cancer, or Tis colorectal cancer that had healed by endoscopic mucosal resection. Women pregnant or lactating at any time during the study period were excluded. Patients were excluded if they had a psychiatric disease or symptoms, were considered unable to participate in the clinical study, had acute myocardial infarction onset within 6 months prior to the protocol surgery, had unstable angina, had high‐grade emphysema or lung fibrosis, were receiving continuous steroids, or were undergoing intersphincteric resection. Other cases were excluded because the doctors considered them ineligible for enrollment in this study.

### 2.4 Definition of facility

In this study, we defined the top three centers in terms of the number of registrations as high‐volume centers and the others as low‐volume centers.

### 2.5 Protocol treatment

The following items were preoperatively defined and registered by the attending surgeon: Preoperative treatment (chemotherapy or radiation therapy) was decided by the attending surgeon, as necessary. Surgery required the participation of a “Board Certified Surgeon in the Japanese Society of Gastroenterological Surgery” as an operator or instructor. The patient underwent anterior resection of the rectum with dissection of the root lymph nodes of the IMA. Reconstruction was performed using DST. If the surgeon planned LCA preservation at registration, the patient was assigned to the “D3 with LCA preservation” group. The approach (conventional laparotomy or laparoscopic surgery) was determined based on the attending surgeon's judgment. Ostomy formation to protect the anastomosis and drain placement were performed at the discretion of the physician in charge. In cases with a coexisting benign disease (gallstone, hernia, etc.) that did not affect the prognosis, surgery could be performed at the same time as surgery for rectal cancer. Informed consent for details of the surgical procedure and written informed consent for this study were obtained from patients prior to surgery. Surgical quality was confirmed by central judgment using images of the surgical fields after anastomosis and the resected specimens. Adjuvant chemotherapy is recommended in patients with stage III disease.

### 2.6 Dissection of lymph nodes at the IMA root

The area of lymph node dissection at the IMA root was defined as the area extending from the IMA root to the LCA bifurcation. En bloc dissection of the tissue between the IMA and the inferior mesenteric vein was performed in accordance with a previous report.^21^ Briefly, the tunica adventitia of the IMA was exposed, and the surrounding tissue was collectively excised by the peri‐arterial tissue of the IMA. Dissection quality was confirmed by central judgment using images of the surgical field (Figure 1A,C). In the central judgment, the root of the inferior mesenteric artery was assessed for adequate exposure and resection of the surrounding fatty tissue (no. 253).

### 2.7 Evaluation

We recorded the patients' background, tumor findings, surgical findings, postoperative course, histopathological findings, and long‐term results. The primary endpoint was the incidence of anastomotic leakage, which was assessed based on clinical symptoms. Briefly, contrast radiography was performed on patients with purulent discharge from the drainage tube or other peritonitis‐related symptoms. The anastomotic leakage was graded as proposed by the International Study Group of Rectal Cancer.^22^ Briefly, grade A required no change in management, grade B required active therapeutic intervention without re‐laparotomy or laparoscopic surgery, and grade C required re‐laparotomy of laparoscopic surgery. Routine radiologic assessment was not performed in asymptomatic patients during the protocol treatment. The secondary endpoints were scheduled surgical completion rate, 3‐year postoperative recurrence‐free survival rate, and exploratory analysis of factors affecting anastomotic leak occurrence. If an adverse event was observed, the best possible action was taken and details were provided in the medical records and case reports.

### 2.8 Adjuvant therapy

The surgeon advised all patients with stage III cancer to undergo postoperative adjuvant chemotherapy by the surgeon in charge. All the patients who provided written informed consent underwent chemotherapy.

### 2.9 Follow‐up schedule

Patients were followed up with outpatient examinations, including tumor marker measurements and chest, abdominal, and pelvic computed tomography (CT) every 6 months for the first 3 years. These examinations were conducted once a year during the fourth and fifth years. Colorectal endoscopy was performed every 2 years. Follow‐up CT images were not assessed using a central judgment.

### 2.10 Statistical analyses

Continuous parameters are presented as mean and standard deviation or median and interquartile range. Fisher's exact test was used in order to compare data between groups for all cohorts. The Kaplan–Meier method was used to estimate survival, and the log‐rank test was used to assess estimated survival. The odds ratio (OR) was estimated using a logistic regression model and the 95% confidence interval (CI) was calculated. Variables were included in the models based on existing knowledge of risk factors for surgical site infection (SSI). Statistical significance was assessed using a 95% CI, and *p* < 0.05 was considered significant. All *p* values were two‐tailed. All data were statistically analyzed using JMP version 15.1.0 (SAS Institute Inc., Cary, NC, USA) and SAS statistical software (version 9.4; SAS Institute Inc.).

To determine the sample size required for this study, we assumed that the incidence of anastomotic leakage in the Japanese D3 without LCA preservation group would be 13% and that preservation of the LCA would reduce the incidence of anastomotic leakage by 3%. Consequently, the number of patients required for this study was estimated to be 800. Because it took approximately 5 years to enroll the 457 cases, an interim analysis was conducted at this point, although this was not specified in the protocol. When 457 patients were enrolled, the leak rate was 7.14% (3.93%–12.56%) in the Japanese D3 without LCA preservation group and 7.86% (4.44%–13.52%) in the Japanese D3 with LCA preservation group. The statistical power at the time of accumulation of 457 cases was 0.08, and even after enrolling up to 800 cases with the same leakage rate, the estimated statistical power was 0.104, which was much lower than originally predicted; therefore, further enrollment was discontinued.

### 2.11 Propensity score matching

The PS model was estimated using a logistic regression model adjusted for age, sex, body mass index, serum albumin level, previous abdominal surgery, history of diabetes mellitus, history of hypertension or hyperlipidemia, bowel obstruction, c‐Stage, tumor location, surgical approach, and tumor size. These variables were chosen based on the clinical knowledge of their potential association with the outcome of interest. PSM was performed according to the resection status and surgical site infection (SSI), mortality, or recurrence risk using nearest‐neighbor matching without replacement, with each patient matched to one control patient, and using calipers of 0.2 width. Standardized differences were used to measure the covariate balance. Twelve variables were used for PSM: age, sex, body mass index (BMI), albumin level, previous abdominal surgery, diabetes, hypertension or hyperlipidemia, bowel obstruction, tumor stage, tumor location, open surgery, and tumor size.

## 3 RESULTS

### 3.1 Background characteristics

**FIGURE 2:**
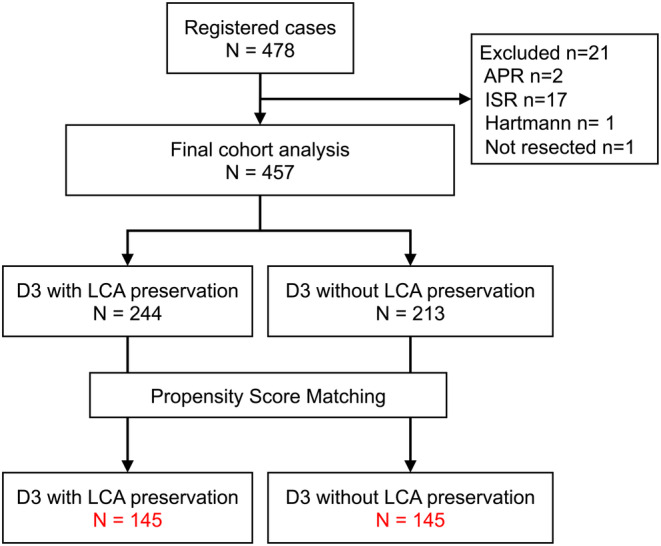
Patient flow diagram. APR indicates abdominoperineal resection; Hartmann, Hartmann procedure; ISR, intrasphincteric resection; LCA, left colic artery.

We enrolled 478 patients with primary CRC between October 1, 2011, and December 31, 2016. Regarding surgical quality, there was no difference in image judgment between the two groups. After exclusion, our analyses included 457 patients who underwent radical resection of rectal cancer. Figure 2 shows a flowchart of patient inclusion and exclusion criteria. Among the included patients, 244 were registered in the mesorectal excision (ME) with LCA preservation group and 213 in the ME without LAC preservation group (Figure 2). D3 with LCA preservation was successfully completed in 218 (89.3%) of the 244 patients registered to this group. All 213 patients in the latter group successfully underwent D3 without LCA preservation. The groups differed significantly in serum albumin levels, rates of previous abdominal surgery, diabetes mellitus, hypertension, laparoscopic surgery, tumor location, and estimated blood loss (Table 1). After PSM, 145 patients in each group were matched; all covariates were balanced, and no statistically significant between‐group differences were observed (Table 1).

**TABLE 1:** Clinical and demographic data from study patients operated for rectal cancer with double‐stapling technique.

	Before matching				After matching			
	D3 with LCA preservation	D3 without LCA preservation	Standardized difference	*p* value	D3 with LCA preservation	D3 without LCA preservation	Standardized difference	*p* value
	*n* = 244	*n* = 213						
					*n* = 145	*n* = 145		
Sex								
Male, *n* (%)	153 (62.7)	131 (61.5)	−0.025	0.792	84 (48.55)	89 (51.45)	0.06	0.5495
Female, *n* (%)	91 (37.3)	82 (38.5)			61 (52.14)	56 (47.86)		
Age, median (IQR)	66.5 (60.0–73.0)	67 (59.5–74.0)	−0.011	0.66	67 (60.0–73.0)	68 (60.5–75.0)	0.01	0.5311
BMI, median (IQR)	22.26 (20.3–24.4)	22.2 (19.9–24.3)	0.009	0.62	22.33 (20.29–24.39)	22.58 (20.06–24.37)	0.07	0.8572
Serum albumin, g/dL, median (IQR)	4.1 (3.8–4.3)	4 (3.6–4.3)	−0.076	0.028	4.1 (3.8–4.4)	4.0 (3.6–4.3)	0.24	0.0884
Previous abdominal surgery, *n* (%)	65 (26.6)	38 (17.8)	0.21	0.025	27 (18.6)	24 (16.6)	0.05	0.6436
DM, *n* (%)	24 (9.7)	40 (18.7)	0.26	0.0060	20 (13.8)	24 (16.6)	0.08	0.5126
HT or HL, *n* (%)	62 (25.4)	85 (39.9)	0.31	0.0009	48 (33.1)	51 (35.2)	0.04	0.7102
Bowel obstruction, *n* (%)	31 (12.7)	27 (12.7)	0.16	0.98	19 (13.1)	15 (10.3)	0.09	0.4653
Stage, *n* (%)								
I	49 (20.1)	39 (18.4)	0.04	0.74	30 (20.6)	31 (21.4)	0.02	0.9712
II	91 (37.3)	75 (35.6)	0.04		53 (36.6)	54 (37.2)	0.01	
III	104 (42.6)	98 (46.2)	0.07		62 (42.8)	60 (41.4)	0.03	
Tumor location, *n* (%)								
Rectosigmoid	72 (29.5)	93 (43.7)	0.30	0.0005	47 (32.4)	52 (35.9)	0.07	0.7928
Upper rectum	112 (45.9)	93 (43.7)	0.04		72 (49.7)	70 (48.3)	0.03	
Lower rectum	60 (24.6)	27 (12.7)	0.31		26 (17.9)	23 (15.9)	0.06	
Approach, *n* (%)								
Laparoscopic surgery	154 (63.1)	192 (90.1)	0.66	<0.0001	125 (86.2)	124 (85.5)	0.02	0.8662
Open surgery	90 (36.9)	21 (9.9)			20 (13.8)	21 (14.5)		
Tumor size, cm, median (IQR)	4 (3.0–5.5)	4.5 (3.0–6.0)	−0.15	0.16	4 (3–5.5)	4 (3–5.5)	0.05	0.6792
Operation time, min, median (IQR)	260.5 (210–349.8)	252 (201–331)	0.18	0.14	266 (210–346)	256 (196.5–340)	0.19	0.2167
Estimated blood loss, g, median (IQR)	70 (10.0–195.0)	27 (10–80)	0.19	0.0003	30.0 (3–120)	30 (10–100)	0.08	0.5019
Ostomy formation	57 (23.4)	36 (16.90)		0.087	27 (18.6)	26 (17.9)	0.02	0.8792
Neoadjuvant therapy, *n* (%)	13 (5.35)	15 (7.04)	0.07	0.4526	10 (6.94)	12 (8.28)	0.04	0.6696
Distance from anal verge, cm, median (IQR)	10 (7–13)	10 (9–14)	0.18	0.0002	10 (7–13)	10 (8–13)	0.07	0.2516

*Note*: Left, unmatched patients (*n* = 457), excluding those with reconstruction mode other than double‐stapling technique (*n* = 21). Right, results from propensity score matching (*n* = 280).

Abbreviations: BMI, body mass index; DM, diabetes mellitus; D3, indicates Japanese D3; HL, hyperlipidemia; HT, hypertension; IQR, interquartile range; LCA, left colic artery; *n*, number; I, II, III, classified with TNM Classification of Malignant tumors, 7th Edition.

### 3.2 Risk factors for anastomotic leak

Table 2 shows the correlation between anastomotic leak occurrence and each factor after PSM. The leakage rates were 8.28% (12/145) after D3 with LCA preservation and 6.90% (10/145) after D3 without LCA preservation, with no significant intergroup differences. After PSM, no analyzed factors were significantly related to the incidence of anastomotic leak, including age, sex, body mass index (BMI), serum albumin level, previous abdominal surgery, diabetes mellitus, hypertension, hyperlipidemia, preoperative ileus, cancer stage, tumor location, operative approach, tumor size, and ostomy creation (Table 2). Our analysis did not indicate that LCA preservation affected the anastomotic leakage grade (Table 3). A subgroup analysis of the factors affecting anastomotic leakage (Table S1) was performed; however, there were no statistically significant factors affecting anastomotic leakage.

**TABLE 2:** Factors for anastomotic leak as the end point in propensity score matched cohort.

		Anastomotic leak			
		Yes (*n* = 22)	No (*n* = 268)	OR (95% CI)	*p* value
D3 with LCA preservation, *n* (%: 95% CI)		12 (8.28: 4.80–13.91)	133 (91.72: 86.09–95.20)	Reference	
D3 without LCA preservation, *n* (%: 95% CI)		10 (6.90: 3.79–12.23)	135 (93.10: 87.77–96.21)	0.82 (0.34–1.96)	0.66
Sex					
Female, *n* (%)		5 (3.70)	112 (95.73)	Reference	
Male, *n* (%)		17 (9.83)	156 (90.17)	2.44 (0.87–6.81)	0.088
Age, median (IQR)		67 (53.75–74.75)	67 (61–74)	0.98 (0.95–1.02)	0.44
BMI, median (IQR)		22.32 (21.22–24.31)	22.48 (20.14–24.39)	1.00 (0.88–1.14)	0.94
Serum albumin, g/dL, median (IQR)		4.1 (3.8–4.4)	4.1 (3.65–4.3)	0.67 (0.27–1.68)	0.40
Previous abdominal surgery, *n* (%)					
No		19 (7.95)	220 (92.05)	Reference	
Yes		3 (5.88)	48 (94.12)	0.72 (0.21–2.54)	0.61
DM, *n* (%)					
No		18 (7.32)	228 (92.68)	Reference	
Yes		4 (9.09)	40 (90.91)	1.27 (0.41–3.94)	0.68
HT or HL, *n* (%)					
No		13 (6.81)	178 (93.19)	Reference	
Yes		9 (9.09)	90 (90.91)	1.37 (0.56–3.32)	0.49
Bowel obstruction, *n* (%)					
No		15 (5.86)	241 (94.14)	Reference	
Yes		7 (20.59)	27 (79.41)	4.17 (1.56–11.11)	0.0044
Stage, *n* (%)					
I		1 (1.64)	60 (98.36)	Reference	
II		10 (7.48)	99 (92.52)	4.85 (0.59–39.73)	0.14
III		10 (10.66)	109 (89.34)	7.16 (0.91–56.04)	0.061
Tumor location, *n* (%)					
Rectosigmoid		6 (6.06)	93 (93.94)	Reference	
Upper rectum		15 (10.56)	127 (89.44)	1.83 (0.68–4.90)	0.23
Lower rectum		1 (2.04)	48 (97.96)	0.32 (0.04–2.76)	0.30
Approach, *n* (%)					
Laparoscopic surgery		20 (8.03)	229 (91.97)	Reference	
Open surgery		2 (4.88)	39 (95.12)	1.70 (0.38–7.58)	0.48
Tumor size, cm, median (IQR)		4 (3–5.5)	5.75 (3–7)	1.22 (1.02–1.46)	0.037
Operation time, min, median (IQR)		258 (204.25–340.5)	288 (216.75–362.75)	1.00 (0.99–1.00)	0.98
Estimated blood loss, g, median (IQR)		30 (6.25–110)	55 (16.25–135)	1.00 (0.99–1.00)	1.00
Ostomy formation					
No		19 (8.02)	218 (91.98)	Reference	
Yes		3 (5.66)	50 (94.34)	0.69 (0.20–2.42)	0.56

Abbreviations: BMI, body mass index; DM, diabetes mellitus; D3, Japanese D3; HL, hyperlipidemia; HT, hypertension; IQR, interquartile range; LCA, left colic artery; *n*, number; OR odds ratio; 95% CI, 95% confidence interval; I, II, III, classified according to the TNM Classification of Malignant Tumors, 7th Edition.

**TABLE 3:** Relationship between preservation of left colic artery and grade of anastomotic leak in propensity score matched cohort.

	Anastomotic leak				
	Yes			No	
	Grade A	Grade B	Grade C		*p* value
D3 with LCA preservation, *n* (%: 95% CI)	0 (0.0: 0.0–0.0)	4 (2.8: 1.1–6.9)	8 (5.6: 2.8–10.5)	133 (92.7: 86.2–95.2)	0.49
D3 without LCA preservation, *n* (%: 95% CI)	1 (0.69: 0.12–3.8)	1 (0.69: 0.12–3.8)	8 (5.5: 2.8–10.5)	135 (93.1: 87.8–96.2)	

Abbreviations: D3, Japanese D3; LCA, left colic artery; *n*, number; 95% CI, 95% confidence interval.

### 3.3 Overall survival and recurrence‐free survival

**FIGURE 3:**
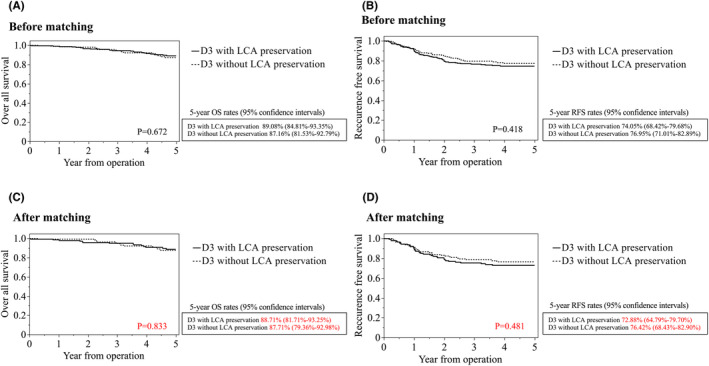
Overall survival (OS) and recurrence‐free survival (RFS) before and after propensity score matching. (A) OS before matching. (B) RFS before matching. (C) OS after propensity score matching. (D) DFS after propensity score matching.

Within the entire cohort, the overall 5‐year survival rates were 89.1% in the D3 with LCA preservation group and 87.2% in the D3 without LCA preservation group. The recurrence‐free survival rates were 74.1% and 77.0%, respectively. After PSM, the overall survival rates were 88.7% and 87.7% with and without LCA preservation, respectively. Recurrence‐free survival rates were 72.9% and 76.4%, respectively (Figure 3).

Table 4 summarizes the risks for overall survival and recurrence‐free risks using propensity score–matched data. LCA preservation did not affect overall survival or recurrence‐free survival. Multivariate analysis revealed that age (HR 1.04, 95% CI 1.00–1.08) and UICC stage (HR 2.63, 95% CI 1.15–1.6.01, stage III relative to stage I or II) were significant risk factors for overall survival. Additionally, sex (HR 1.76, 95% CI 1.04–2.98), preoperative bowel obstruction (HR 1.75, 95% CI 0.95–3.21), and UICC stage (HR 2.38, 95% CI 1.43–3.94, stage III relative to stage I or II) were significant risk factors for recurrence‐free survival.

**TABLE 4:** Multivariate analysis of overall survival and recurrence‐free survival using the propensity score matching dataset.

		OS				RFS			
		Univariate analysis		Multivariate analysis		Univariate analysis		Multivariate analysis	
		HR (95% CI)	*p* value	HR (95% CI)	*p* value	HR (95% CI)	*p* value	HR (95% CI)	*p* value
D3 with LCA preservation		Reference		Reference		Reference		Reference	
D3 without LCA preservation		1.10 (0.51–2.30)	0.84	1.10 (0.51–2.39)	0.80	0.84 (0.53–1.4)	0.48	0.81 (0.51–1.31)	0.390
Sex									
Female		Reference		Reference		Reference		Reference	
Male		1.36 (0.61–3.03)	0.45	1.12 (0.49–2.56)	0.796	1.78 (1.06–2.99)	0.030	1.76 (1.04–2.98)	0.0343
Age		1.04 (0.99–1.1)	0.054	1.04 (1.00–1.08)	0.038	1.01 (0.9–1.03)	0.29		
BMI		1.08 (0.97–1.20)	0.15			1.02 (0.95–1.09)	0.61		
Serum albumin, g/dL		0.50 (0.24–1.10)	0.085			0.75 (0.46–1.26)	0.28		
Previous abdominal surgery									
Absence		Reference				Reference			
Presence		0.61 (0.18–2.02)	0.42			0.79 (0.41–1.55)	0.50		
DM									
Absence		Reference		Reference		Reference			
Presence		2.74 (1.23–6.09)	0.014	2.20 (0.96–5.03)	0.063	1.48 (0.82–2.66)	0.19		
HT or HL									
Absence		Reference				Reference			
Presence		1.54 (0.72–3.28)	0.27			0.98 (0.59–1.61)	0.93		
Bowel obstruction									
Absence		Reference				Reference		Reference	
Presence		0.88 (0.26–2.91)	0.83			2.30 (1.28–4.14)	0.0054	1.75 (0.95–3.21)	0.0343
Stage UICC									
I or II		Reference		Reference		Reference		Reference	
III		2.73 (1.23–6.08)	0.014	2.63 (1.15–6.01)	0.0219	2.55 (1.57–4.15)	<0.001	2.38 (1.43–3.94)	0.001
Tumor location									
Rectosigmoid		Reference				Reference			
Upper rectum		1.70 (0.71–4.08)	0.23			0.95 (0.57–1.58)	0.85		
Lower rectum		0.55 (0.11–2.63)	0.45			0.60 (0.27–1.33)	0.21		
Approach									
Laparoscopic surgery		Reference				Reference			
Open surgery		1.34 (0.40–4.44)	0.64			0.67 (0.37–1.23)	0.20		
Tumor size, cm		1.09 (0.91–1.28)	0.34			1.10 (0.99–1.21)	0.066		
Operation time, min		1.00 (0.99–1.00)	0.22			0.99 (0.99–1.00)	0.97		
Estimated blood loss, g		1.00 (1.00–1.00)	0.019			1.00 (0.99–1.00)	0.073		
Ostomy formation									
Absence		Reference				Reference			
Presence		1.34 (0.54–3.31)	0.53			0.73 (0.37–1.43)	0.36		

Abbreviations: BMI, body mass index; DM, diabetes mellitus; D3, Japanese D3; HL, hyperlipidemia; HR, hazard ratio; HT, hypertension; IQR, interquartile range; LCA, left colic artery; n, number; OS, indicates overall survival; RFS, recurrence‐free survival; 95% CI, 95% confidence interval; I, II, III, classified according to the TNM Classification of Malignant Tumors, 7th Edition.

### 3.4 LCA preservation success rate

The overall success rate of LCA preservation was 89.0% (218/244). It was 94.8% (128/135) in the high‐volume centers and 82.6% (90/109) in the low‐volume centers (*p* = 0.0029, Table 5).

**TABLE 5:** LCA preservation success rate in all cases (*n* = 478).

	LCA preservation		
	Successful	Failed	*p* value
Overall, *n* (%: 95% CI)	218 (89.34: 84.85–92.62)	26 (10.66: 7.38–15.16)	
High volume center, *n* (%: 95% CI)	128 (94.81: 89.69–97.47)	7 (5.19: 2.53–10.32)	0.0029
Low volume center, *n* (%: 95% CI)	90 (82.57: 74.37–88.55)	19 (17.43: 11.45–ß25.63)	

Abbreviation: LCA, left colic artery.

### 3.5 Number of retrieved lymph nodes

The median number of retrieved lymph nodes was 18 (IQR; 13–22.5) in the D3 with LCA preservation group and 19 (IQR; 13–26) in the D3 without LCA group. There were no statistically significant differences between the two groups (Table S2).

## 4 DISCUSSION

This multicenter, interventional, non‐randomized, open‐label prospective study did not demonstrate any difference in the anastomotic leak rate, overall, and recurrence‐free survival between Japanese D3 with LCA preservation and Japanese D3 without LCA preservation, even after PSM. Only bowel obstruction and tumor size significantly affected anastomotic leakage in the present results.

The primary endpoint was the incidence of anastomotic leakage. The leakage rate was 7.14% (3.93–12.56%) in the Japanese D3 without LCA preservation group and 7.86% (4.44–13.52%) in the Japanese D3 with LCA preservation group. The results of this analysis were obtained by including cases that failed to preserve the LCA in the preservation group; however, the actual success rate of LCA preservation was 89.3%. To corroborate these results, an analysis was performed separately for the groups that were able to preserve the LCA and those that were not, and the results were comparable to the original data. (data not shown). Although our sample size estimate is consistent with the approximately 13% rate of grade B + C anastomotic leakage reported in a prospective multicenter large cohort study (*n* = 1014),^23^ the results of this study showed a lower incidence of suture failure than originally expected. However, the interim analysis of this study showed that the actual incidence of suture failure was 7–8%, which was lower than expected, and it was inferred that accumulating the planned number of patients would not yield significant results; therefore, the study was terminated. It is not known whether this was due to the unification of procedures, such as specifying the surgeon or instructor and submitting the postoperative field of view, or was simply related to selection bias in clinical trials.

The major strength of this study was that it was based on a preoperative registration‐based trial, which helped limit the loss of detailed and follow‐up data. To overcome selection bias, we used PSM and other relevant modern statistical methods in consultation with a statistician (M.F.), with the aim of minimizing bias and residual confounding. By limiting the patients to cT2 or deeper, this study included only those who required Japanese D3 dissection. To ensure high‐quality surgery, operations require the participation of a board‐certified surgeon in gastroenterological surgery as an operator or instructor. The quality of lymph node dissection was confirmed by central judgment using images of the surgical field. Notably, the most original aspect of this study was that lymph node dissection levels were common between the two groups; therefore, we compared only the tie levels of the IMA. In addition, the patients represented a well‐balanced ratio of those who underwent open surgery to those who underwent laparoscopic surgery.

One limitation of this study was its non‐randomized design. Additionally, it was stopped prematurely because the registration was slower than expected. This may have introduced bias, although it is unlikely that including the number of patients assumed in the power calculation would have changed the most important results since the difference between the two groups was small. Notably, it appears impossible to achieve a trial large enough to detect modest differences between the tie levels. Regarding the assessment of anastomotic leakage, because routine radiologic assessment was not performed in asymptomatic patients during protocol treatment, the incidence of leakage may have been underestimated, especially in patients with ostomy formation. Also, at the time of creating the protocol at the beginning of the study, it was generally believed that mobilization of the splenic flexure was not necessary for the Japanese body type, so data regarding the presence or absence of mobilization of the splenic flexure were not sampled.

The success rate of the LCA preservation was 89.3%; however, we could not determine whether the procedure was easy to perform. In a sub‐analysis of this study, the LCA preservation success rate was 94.8% at the high‐volume center, whereas it was statistically lower at other facilities. It is possible that the technical disparity between facilities was a confounding factor in our results. It should be noted that we observed a disparity between facilities in the success rate of LCA preservation, although the procedures at all facilities included the participation of a board‐certified surgeon in gastroenterological surgery. The development of robotic surgery has made complex procedures possible. Preserving the LCA without preserving the IMA root^24^ may yield different results because it reduces tension on the oral colon stump, which is a weakness of conventional LCA preservation methods. Emerging evidence suggests that blood perfusion at the intestinal stump affects the development of anastomotic insufficiency,^25^ and LCA preservation may still be a useful option in patients with a strong predisposition to arteriosclerosis, such as those with severe diabetes and hypertension.^26^, ^27^, ^28^ In a sub‐analysis of JCOG0404, a Japanese randomized controlled trial, LCA preservation was not associated with the leak rate in sigmoid colon cancer and upper rectal cancer, but was associated with short‐term outcomes. LCA preservation is not associated with leak rate but is positively associated with short‐ and long‐term outcomes for sigmoid colon cancer and upper rectal cancer.^29^

In conclusion, our findings suggest that LCA preservation does not affect the incidence of anastomotic leakage after rectal resection with anastomosis using DST, and may not affect oncological outcomes. Notably, the facilities differed in the degree of training in the technique for LCA preservation, and it may be more desirable for LCA preservation to be performed at high‐volume centers.

## AUTHOR CONTRIBUTIONS

**Hidekazu Takahashi:** Conceptualization; data curation; formal analysis; funding acquisition; investigation; methodology; project administration; validation; visualization; writing – original draft; writing – review and editing. **Kazuhiro Saso:** Conceptualization; data curation; formal analysis. **Masayuki Ohue:** Conceptualization; formal analysis; investigation. **Shingo Noura:** Conceptualization; investigation; methodology; supervision. **Tsukasa Tanida:** Data curation; project administration; validation. **Takamichi Komori:** Conceptualization; investigation; supervision. **Mitsuyoshi Tei:** Formal analysis; methodology. **Yoshinori Kagawa:** Data curation. **Shunji Morita:** Data curation; formal analysis. **Shu Okamura:** Data curation; methodology. **Masakazu Miyake:** Conceptualization; software; supervision; validation. **Norikatsu Miyoshi:** Resources; software; supervision. **Mamoru Uemura:** Conceptualization; resources; software; supervision. **Makoto Fujii:** Formal analysis; methodology; visualization. **Yuko Ohno:** Conceptualization; formal analysis; methodology; supervision; validation. **Hirofumi Yamamoto:** Formal analysis; supervision; validation. **Kohei Murata:** Supervision. **Yuichiro Doki:** Supervision. **Hidetoshi Eguchi:** Supervision.

## CONFLICT OF INTEREST STATEMENT

Yuichiro Doki is an editorial board member of *Annals of Gastroenterological Surgery*.

## ETHICS STATEMENT

Approval of the research protocol by an Institutional Reviewer Board: This study was approved by the Ethics Review Committee of Osaka University (11105) and conforms to the provisions of the Declaration of Helsinki.

Informed Consent: Written informed consent was obtained from all patients before enrollment.

Registry and the Registration No. of the study/trial: This study was registered with UMIN‐CTR (UMIN000006160).

Animal Studies: N/A.

## Supporting information


Tables S1.

## References

[1] Dekker E , Tanis PJ , Vleugels JLA , Kasi PM , Wallace MB . Colorectal cancer. Lancet. 2019;394(10207):1467–1480. 31631858 10.1016/S0140-6736(19)32319-0

[2] Siegel RL , Miller KD , Jemal A . Cancer statistics, 2018. CA Cancer J Clin. 2018;68(1):7–30. 29313949 10.3322/caac.21442

[3] Ferlay J , Shin HR , Bray F , Forman D , Mathers C , Parkin DM . Estimates of worldwide burden of cancer in 2008: GLOBOCAN 2008. Int J Cancer. 2010;127:2893–2917. 21351269 10.1002/ijc.25516

[4] Heald RJ , Husband EM , Ryall RD . The mesorectum in rectal cancer surgery—the clue to pelvic recurrence? Br J Surg. 1982;69(10):613–616. 6751457 10.1002/bjs.1800691019

[5] Hohenberger W , Weber K , Matzel K , Papadopoulos T , Merkel S . Standardized surgery for colonic cancer: complete mesocolic excision and central ligation—technical notes and outcome. Color Dis. 2009;11(4):354–364. 10.1111/j.1463-1318.2008.01735.x 19016817

[6] West NP , Hohenberger W , Weber K , Perrakis A , Finan PJ , Quirke P . Complete mesocolic excision with central vascular ligation produces an oncologically superior specimen compared with standard surgery for carcinoma of the colon. J Clin Oncol. 2010;28(2):272–278. 19949013 10.1200/JCO.2009.24.1448

[7] Bertelsen CA , Neuenschwander AU , Jansen JE , Wilhelmsen M , Kirkegaard‐Klitbo A , Tenma JR , et al. Disease‐free survival after complete mesocolic excision compared with conventional colon cancer surgery: a retrospective, population‐based study. Lancet Oncol. 2015;16(2):161–168. 25555421 10.1016/S1470-2045(14)71168-4

[8] West NP , Kobayashi H , Takahashi K , Perrakis A , Weber K , Hohenberger W , et al. Understanding optimal colonic cancer surgery: comparison of Japanese D3 resection and European complete mesocolic excision with central vascular ligation. J Clin Oncol. 2012;30(15):1763–1769. 22473170 10.1200/JCO.2011.38.3992

[9] Brierley JD , Gospodarowics MK , Wittekind C . TNM classification of malignant tumors. 7th ed. Hoboken NJ: John Wiley & Sons Ltd; 2009.

[10] Dukes CE . The spread of cancer of the rectum. Br J Surg. 1930;17:643.

[11] Mc EJ , Bacon HE , Trimpi HD . Lymph node metastases; experience with aortic ligation of inferior mesentery artery in cancer of the rectum. Surgery. 1954;35(4):513–531. 13156863

[12] Kanemitsu Y , Hirai T , Komori K , Kato T . Survival benefit of high ligation of the inferior mesenteric artery in sigmoid colon or rectal cancer surgery. Br J Surg. 2006;93(5):609–615. 16607682 10.1002/bjs.5327

[13] Alici A , Kement M , Gezen C , Akın T , Vural S , Okkabaz N , et al. Apical lymph nodes at the root of the inferior mesenteric artery in distal colorectal cancer: an analysis of the risk of tumor involvement and the impact of high ligation on anastomotic integrity. Tech Coloproctol. 2010;14(1):1–8. 20066459 10.1007/s10151-009-0547-6

[14] Kessler H , Hohenberger W . Extended lymphadenectomy in colon cancer is crucial. World J Surg. 2013;37(8):1789–1798. 23754141 10.1007/s00268-013-2130-6

[15] Charan I , Kapoor A , Singhal MK , Jagawat N , Bhavsar D , Jain V , et al. High ligation of inferior mesenteric artery in left colonic and rectal cancers: lymph node yield and survival benefit. Indian J Surg. 2015;77:1103–1108. 27011519 10.1007/s12262-014-1179-2 PMC4775673

[16] Dworkin MJ , Allen‐Mersh TG . Effect of inferior mesenteric artery ligation on blood flow in the marginal artery‐dependent sigmoid colon. J Am Coll Surg. 1996;183(4):357–360. 8843265

[17] Lange MM , Buunen M , van de Velde CJH , Lange JF. Level of arterial ligation in rectal cancer surgery: low tie preferred over high tie. A review. Dis Colon Rectum. 2008;51:1139–1145. 18483828 10.1007/s10350-008-9328-y PMC2468314

[18] Komen N , Slieker J , de Kort P , de Wilt JHW , van der Harst E , Coene PP , et al. High tie versus low tie in rectal surgery: comparison of anastomotic perfusion. Int J Color Dis. 2011;26(8):1075–1078. 10.1007/s00384-011-1188-6 PMC3140934 21445553

[19] Bonnet S , Berger A , Hentati N , Abid B , Chevallier JM , Wind P , et al. High tie versus low tie vascular ligation of the inferior mesenteric artery in colorectal cancer surgery: impact on the gain in colon length and implications on the feasibility of anastomoses. Dis Colon Rectum. 2012;55:515–521. 22513429 10.1097/DCR.0b013e318246f1a2

[20] Luo Y , Li R , Wu D , Zeng J , Wang J , Chen X , et al. Long‐term oncological outcomes of low anterior resection for rectal cacer with and without preservation of the left colic artery: a retrospective cohort study. BMC Cancer. 2021;21:171. 33596860 10.1186/s12885-021-07848-y PMC7890901

[21] Sekimoto M , Takemasa I , Mizushima T , Ikeda M , Yamamoto H , Doki Y , et al. Laparoscopic lymph node dissection around the inferior mesenteric artery with preservation of the left colic artery. Surg Endosc. 2011;25(3):861–866. 20725744 10.1007/s00464-010-1284-7

[22] Rahbari NN , Weitz J , Hohenberger W , Heald RJ , Moran B , Ulrich A , et al. Definition and grading of anastomotic leakage following anterior resection of the rectum: a proposal by the International Study Group of Rectal Cancer. Surgery. 2010;147(3):339–351. 20004450 10.1016/j.surg.2009.10.012

[23] Shiomi A , Ito M , Maeda K , Kinugasa Y , Ota M , Yamaue H , et al. Effects of a diverting stoma on symptomatic anastomotic leakage after low anterior resection for rectal cancer: a propensity score matching analysis of 1,014 consecutive patients. J Am Coll Surg. 2015;220(2):186–194. 25529899 10.1016/j.jamcollsurg.2014.10.017

[24] Malakorn S , Sammour T , Bednarski B , You YQN , Chang GJ . Three different approach to the inferior mesenteric artery during robotic D3 lymphadenectomy for rectal cancer. Ann Surg Oncol. 2017;24(7):1923. 28213788 10.1245/s10434-017-5792-8

[25] Emile SH , Khan SM , Wexner SD . Impact of change in the surgical plan based on indocyanine green fluorescence angiography on the ratas of colorectal anastomosis leak: a systematic review and meta‐analysis. Surg Endsc. 2022;36:2245–2257. 10.1007/s00464-021-08973-2 35024926

[26] Fawcett A , Shembekar M , Church JS , Vashisht R , Springall RG , Nott DM . Smoking, hypertension, and colonic anastomotic healing; a combined clinical and histopathological study. Gut. 1996;38(5):714–718. 8707117 10.1136/gut.38.5.714 PMC1383153

[27] Kingham PT , Pachter LH . Colonic anastomotic leak: risk factors, diagnosis, and treatment. J Am Coll Surg. 2009;208(2):269–278. 19228539 10.1016/j.jamcollsurg.2008.10.015

[28] Huisman DE , Reudink M , van Rooijen SJ , Bootsma BT , van de Brug T , Stens J , et al. LekCheck: a prospective study to identify perioperative modiciable risk factor for anastomotic leakage in colorectal surgery. Ann Surg. 2022;275(1):e189–e197. 32511133 10.1097/SLA.0000000000003853 PMC8683256

[29] Akagi T , Inomata M , Hara T , Mizusawa J , Katayama H , Shida D , et al. Clinical impact of D3 lymph node dissection with left colic artery (LCA) preservation compared to D3 without LCA preservation: exploratory subgroup analysis of data from JCOG0404. Ann Gastroenterol Surg. 2020;4(2):163–169. 32258982 10.1002/ags3.12318 PMC7105844

