# Supplementary material for: Efficacy of lymph node dissection around the inferior mesenteric artery with preservation of the left colic artery for rectal cancer
Source: Ann Gastroenterol Surg. 2024 Oct 11;9(2):298–308. doi: 10.1002/ags3.12869 (PMC11877326; doi:10.1002/ags3.12869)
Supplement: Supplementary file 1 — Tables S1. [file AGS3-9-298-s001.docx]

**Supplementary Table 1.** Subgroup analysis of factors affecting anastomotic leakage

|  | |  | Patients with anastomotic leakage, n (%: 95% CI) | Number of patients without anastomotic leakage, n (%: 95% CI) | *p*-value |
| --- | --- | --- | --- | --- | --- |
| All cases |  | D3 with LCA preservation | 12 (8.28: 4.80–13.91) | 133 (91.72: 86.09–95.20) | 0.6700 |
|  |  | D3 without LCA preservation, | 10 (6.90: 3.79–12.23) | 135 (93.10: 87.77–96.21) |  |
| DM | yes | D3 with LCA preservation | 8 (6.40: 3.28–12.12) | 117 (93.60: 87.88–96.72) | 0.5890 |
|  |  | D3 without LCA preservation | 10 (8.26: 4.55–14.55) | 111 (91.74: 85.45–95.45) |  |
|  | no | D3 with LCA preservation | 4 (20.00: 8.07–ß41.60) | 16 (80.00: 58.40–91.93) | 0.0515 |
|  |  | D3 without LCA preservation | 0 (0.00) | 24 (100.00) |  |
| HT or HL | yes | D3 with LCA preservation | 7 (7.22: 3.54–14.15) | 90 (92.78: 85.85–96.46) | 0.8361 |
|  |  | D3 without LCA preservation | 6 (6.38: 2.96–13.23) | 88 (93.62: 86.77–97.04) |  |
|  | no | D3 with LCA preservation | 5 (10.42: 4.53–22.17) | 43 (89.58: 77.83–95.47) | 0.6741 |
|  |  | D3 without LCA preservation | 4 (7.84: 3.09–18.50) | 47 (92.16: 81.50–96.91) |  |
| Distance from anal verge | <10 cm | D3 with LCA preservation | 2 (3.39: 0.93–11.54) | 57 (96.61: 88.46–99.07) | 0.1070 |
|  |  | D3 without LCA preservation | 6 (12.50: 5.86–24.70) | 42 (87.50: 75.30–94.14) |  |
|  | ≥10 cm | D3 with LCA preservation | 10 (11.63: 6.44–20.10) | 76 (88.37: 79.90–93.56) | 0.0731 |
|  |  | D3 without LCA preservation | 4 (4.12: 1.62–10.13) | 93 (95.88: 89.87–98.38) |  |
| Tumor size | <4.3 cm | D3 with LCA preservation | 3 (3.90­: 1.33–10.84) | 74 (96.10: 89.16­–98.67) | 0.6872 |
|  |  | D3 without LCA preservation | 4 (5.41: 2.12­–13.09) | 70 (94.60­: 86.91–97.88) |  |
|  | ≥4.3 cm | D3 with LCA preservation | 9 (13.24: 7.12–23.28) | 59 (86.77: 76.72–92.88) | 0.3861 |
|  |  | D3 without LCA preservation | 6 (8.45: 3.93–17.24) | 65 (91.55: 82.76–96.07) |  |
| Stage | I | D3 with LCA preservation | 0 (0.00) | 30 (100.00) | 0.5700 |
|  |  | D3 without LCA preservation | 1 (3.22: 0.57–16.19) | 30 (96.78: 83.81–99.43) |  |
|  | II | D3 with LCA preservation | 4 (7.55: 2.97–17.86) | 49 (92.45: 82.14–97.03) | 0.9762 |
|  |  | D3 without LCA preservation | 4 (7.41: 2.92–17.55) | 50 (92.59: 82.45–97.08) |  |
|  | III | D3 with LCA preservation | 5 (8.33: 3.61–18.07) | 55 (91.67: 81.93–96.39) | 0.4451 |
|  |  | D3 without LCA preservation | 8 (12.90: 6.69–23.45) | 54 (87.10: 76.55–93.31) |  |

n indicates number; 95% CI, 95% confidence interval; D3, Japanese D3; LCA, left colic artery; DM, diabetes mellitus; HT, hypertension; HL, hyperlipidemia; I, II, III, classified according to the TNM Classification of Malignant tumors, 7th Edition.

**Supplementary TABLE 2. Number of retrieved lymph nodes in propensity score matched cohort**

|  | D3 with LCA preservation | D3 without LCA preservation | SD | *P* value |
| --- | --- | --- | --- | --- |
|  | n = 145 | n = 145 |  |  |
| Number of retrieved lymph nodes, median (IQR) | 18 (13–22.5) | 19 (13–26) | 0.09 | 0.1838 |

n, indicates number; D3, Japanese D3; LCA, left colic artery; IQR, interquartile range; SD, standard difference
